# Surfaces of VO_2_‐Polymorphs: Structure, Stability and the Effect of Doping

**DOI:** 10.1002/cphc.202000969

**Published:** 2021-05-04

**Authors:** Berenike Stahl, Thomas Bredow

**Affiliations:** ^1^ Mulliken Center for Theoretical Chemistry Institute for Physical and Theoretical Chemistry University of Bonn Beringstr. 4 D-53115 Bonn Germany; ^2^ MPI for chemical energy conversion Stiftstrasse 34–36 Mülheim an der Ruhr Germany

## Abstract

Vanadium dioxide is an interesting and frequently applied material due to its metal‐insulator phase transition. However, there are only few studies of the catalytic activity and surface properties of different VO_2_ polymorphs. Therefore, we investigated the properties of the surfaces of the most stable VO_2_ phases theoretically at density‐functional theory level using a self‐consistent hybrid functional which has demonstrated its accuracy for the prediction of structural, electronic and energetic properties in a previous study. We found that the surfaces of the rutile R phase of VO_2_ are not stable and show a spontaneous phase transition to the monoclinic M_1_ phase. Doping with Mo stabilizes the surfaces with rutile structure even for small dopant concentrations (6.25 %). Both M_1_ and R surfaces strongly relax, with and without doping. In particular the metal‐metal distances in the uppermost layers change by up to 0.4 Å. Mo segregates in the topmost layer of both R and M_1_ phases. The electronic structure is only slightly changed upon doping.

## Graphical abstract

**Polymorph properties**: The properties of the surfaces of two VO_2_‐polymorphs are investigated using the self‐consisting hybrid method sc‐PBE0. Additionally, the effect of doping on the properties of the polymorph surfaces was assessed by introducing Mo in various positions in the surface models. The V−V distances emerged as an important property in differentiating the polymorphs.

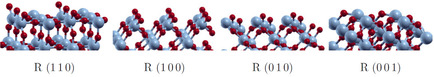

## 1 Introduction

Heterogeneous catalysis faces many challenges in optimizing processes for the overall higher energy demand and need for more sustainable resources. One approach to optimize these processes is to exploit fluctuating reaction conditions.^[1]^ The present study will form the basis of a theoretical study of a particular example, the temperature‐controlled phase change in vanadium dioxide. Vanadium dioxide, VO_2_, is a widely studied compound because of its low temperature metal‐insulator transition which can be exploited in applications such as smart windows,^[2]^ field‐effect transistors or memory devices.^[3]^ At 340 K VO_2_ is transformed from a monoclinic semiconductor (M_1_ phase) to a metallic phase with rutile structure (R phase).^[4]^ The heat of transition from R to M_1_ is −0.044 eV.^[5]^ Simultaneously to the structural and electronic changes during the transition the magnetic state changes.^[6]^ The magnetic ground state of M_1_ phase has been controversial for a long time.^[7]^ Experimentally temperature independent behavior was found.^[6]^ The ground state of the phase was also proposed to be paramagnetic^[8]^. EPR studies and other experimental results indicated electronic correlation to be present in the phase.[^9^, ^10^] Diffusion quantum Monte Carlo found the M_1_ phase to have an antiferromagnetic ground state.^[11]^ The rutile phase has a paramagnetic spin state.[^6^, ^10^, ^11^]

Heterovalent dopants such as Fe, Co, Ni, Mo and W[^12^, ^13^] or oxygen vacancies^[14]^ stabilize the rutile phase and decrease the M_1_‐R phase transition temperature. Isovalent dopants such as Ge and Ti have the opposite effect. According to the literature, W is the most effective dopant atom. It can reduce the transition temperature by up to 27 K per atomic percent.^[13]^ Mo is likewise a promising dopant since it is able to reduce the transition temperature by 5 K/at.%.

Different from W, doping with Mo does not lead to significant changes of the M_1_ lattice structure.[^15^, ^16^, ^17^] Additionally, doping with Mo has been found to assist the synthesis of VO_2_.^[16]^ We therefore decided to use Mo as a dopant in the present study.

Due to their interesting redox chemistry, many vanadium compounds have been investigated as catalyst materials.^[18]^ Among them, also VO_2_ has been found to be catalytically active, e. g. for the desulfurization of dibenzothiophene,^[19]^ the oxidative dehydrogenation of propane^[20]^ or the electrochemical reduction of trinitrotoluene.^[21]^ The effect of the M_1_ →R phase transition in the catalytic oxidative desulfurization of dibenzothiophene has been investigated experimentally.^[19]^ This study gives an indication that the phase transition can be exploited to optimize catalytic processes.

**Figure 1:**
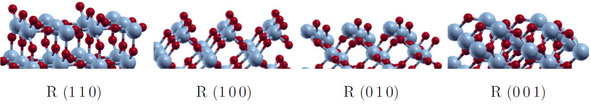
Low‐index surfaces of the rutile phase.

**Figure 2:**
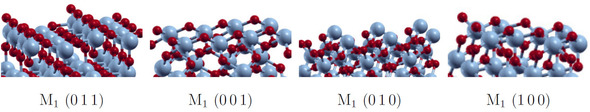
Low‐index surfaces of the monoclinic phase.

The stable surfaces of the M_1_ phase are (0 1 1), (0 0 1), (0 1 0) and (1 0 0). The symmetry‐equivalent surfaces of the R phase are (1 1 0), (1 0 0), (0 1 0) and (0 0 1), respectively. These surfaces have been studied experimentally[^22^, ^23^] and theoretically.[^24^, ^25^, ^26^, ^27^, ^28^] The surface structures are shown in Figure 1 and Figure 2. In most of the previous theoretical work, the focus was only on one of the phases.[^24^, ^25^, ^27^] Here we want to study the properties of all low‐index surfaces of both phases at the same theoretical level. In addition the effect of Mo doping on the relative stability of the two phases and their surfaces is investigated.

## Computational Details

In a previous study^[29]^ we found that a self‐consistent hybrid functional (sc‐PBE0) with 12.7 % Fock‐exchange provides accurate structural, energetic and electronic properties for both VO_2_ phases in the bulk. Therefore, the sc‐PBE0 functional, as implemented the program CRYSTAL17 v1.0.2,^[30]^ is used in this study to calculate surface properties. Comparatively small basis sets with respect to the standard pob‐TZVP basis sets,^[31]^ which were applied in our previous studies on VO_2_,[^7^, ^29^] are used to reduce the computational effort. For Vanadium a modified 86‐411d31G basis set by Harrison et al.^[32]^ is chosen and a 8‐411G(d11) basis set by Heifets et al.^[33]^ is used for oxygen. For the dopant Mo‐atoms a pob‐TZVP basis set is applied^[31]^. The integral truncation tolerances are set to the recommended values for hybrid methods (10^−7^, 10^−7^, 10^−7^, 10^−14^, 10^−42^). A Monkhorst‐Pack net with 4×4×1 k‐points is applied. The models are calculated in a ferromagnetic state in order to increase the symmetry and reduce the computational effort while still taking into account the open‐shell character of VO_2_. Instead of the experimental lattice parameters a=5.743 Å, b=4.517 Å, c=5.375 Å and $\beta =122.6{\;}^{\circ }$ for the M_1_ phase^[34]^ and a=b=4.552 Å and c=2.851 Å for the R phase,^[35]^ the optimized bulk lattice constants were applied for the construction of surface models. These are a=5.967 Å, b=4.590 Å, c=5.310 Å and $\beta =123.4{\;}^{\circ }$ for the M_1_ phase and a=b=4.559 Å and c=2.839 Å for the R phase. Most of the calculated lattice parameters are in good agreement with experiment. The larger deviations compared to our previous study are due to the smaller basis sets.

The surface energy *E_s_* is calculated as

(1) $$E_{s}\left(n\right)=\frac{E_{\mathrm{slab}}\left(n\right)-nE_{\mathrm{bulk}}}{2A}$$

with *n* being the number of stoichiometric layers and *E*_slab_ the total energy of the slab model, *E*_bulk_ the total energy of the bulk and *A* the area of the unit cell. In order to calculate the effect of doping on the surface stability, segregation energies *E*_seg_ are calculated as defined by Alfredsson et al.^[36]^

(2) $$E_{\mathrm{seg}}\left(m\right)=\left(mE_{\mathrm{bulk},\;\mathrm{doped}}+E_{\mathrm{slab}}\right)-\left(mE_{\mathrm{bulk}}+E_{\mathrm{slab},\;\mathrm{doped}}\right)$$

with *m* being the number of dopant atoms in a slab model. The segregation energy *E*_seg_ is added to *E*_slab_ to calculate the surface energy of the doped surface.

For the reference energies 2×2×2 bulk supercells were calculated, which yielded the lattice parameters a=c=4.553 Å and c=2.876 Å for the R phase and a=5.752 Å, b=4.553 Å, c=5.386 and $\beta =122.3{\;}^{\circ }$ for the M_1_ phase.

## 2 Results

### 2.1 Undoped VO_2_

**Figure 3:**
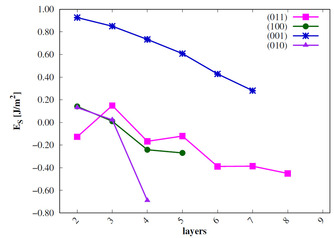
Surface energies of low‐index R surfaces in J/m^2^; sc‐PBE0 results.

**Figure 4:**
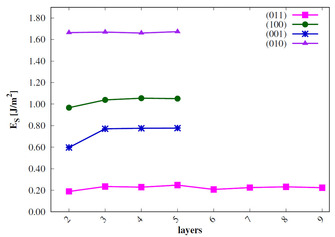
Surface energies of low‐index M_1_ surfaces in J/m^2^; sc‐PBE0 results.

The surface energies *E_s_* are calculated for all low‐index surfaces of the two VO_2_ phases^[23]^ and compared to experimental results. For this purpose, *E_s_* needs to be converged with the number of stoichiometric layers (*n*). The results are shown in Figure 3 and Figure 4.

The surface energies of the monoclinic phase are converged already for n=4 . The calculated *E_s_* of the M $\left(0\;1\;1\right)$ (and also the R $\left(1\;1\;0\right)$ ) surfaces show an odd‐even oscillation as observed for rutile TiO_2_.^[37]^ The order of stability of the M_1_ surfaces is $\left(0\;1\;1\right)>\left(0\;0\;1\right)>\left(1\;0\;0\right)>\left(0\;1\;0\right)$ . This is in agreement with the experimental results[^22^, ^23^] and with previous theoretical studies.^[24]^

**Figure 5:**
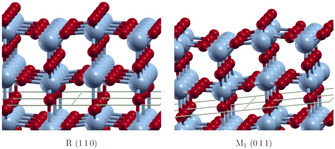
Relaxed structures of the R $\left(1\;1\;0\right)$ and M_1_ $\left(0\;1\;1\right)$ surfaces.

The surface energies calculated for the R phase surfaces do not converge. Furthermore, negative surface energies are obtained or can be expected for larger number of layers. These results indicate that the R surfaces reconstruct and at least partially transform to the M_1_ structure. Since the primitive surface cells do not allow for a V−V bond length alternation, we increased the size of the unit cells in order to analyze the phase transition. For the R $\left(1\;1\;0\right)$ and R $\left(0\;0\;1\right)$ surfaces a $\left(2\times 1\right)$ supercell is used, while the $\left(0\;1\;0\right)$ and $\left(1\;0\;0\right)$ surfaces required a $\left(2\times 2\right)$ supercell. In Figure 5 it can be seen that the relaxed structures e. g. of the R $\left(1\;1\;0\right)$ and M_1_ $\left(0\;1\;1\right)$ surfaces are similar.

**Figure 6:**
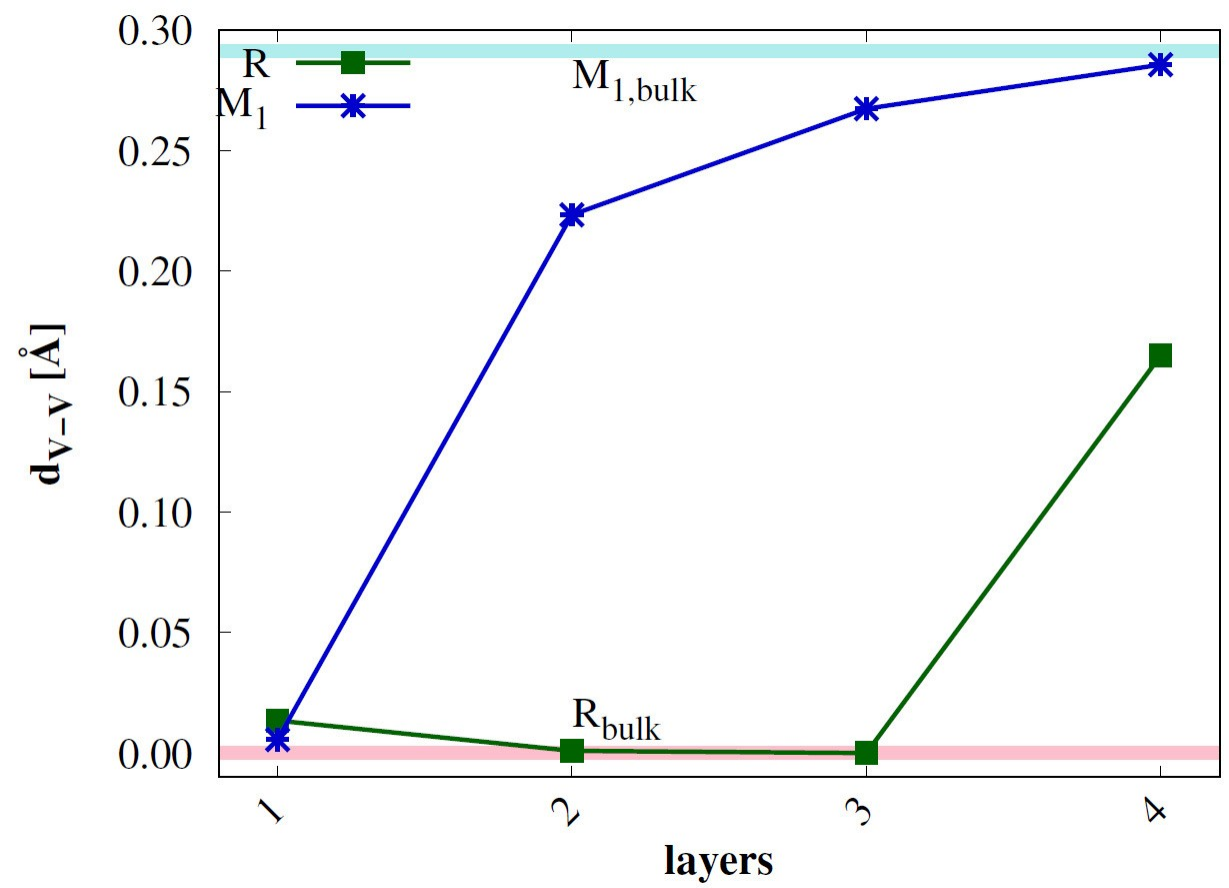
Difference of V−V‐distances d_*V*–*V*_ of the 8 layer R $\left(1\;1\;0\right)$ and M_1_ $\left(0\;1\;1\right)$ surfaces with the number of layers compared to a 2×2×2 bulk R (pink) and M_1_ (light blue) supercell.

Since the main difference between the M_1_ and R bulk structures is the V−V bond alternation in the quasi‐linear V−V chains, the difference of the V−V distances (d_*V*–*V*_) within the V−V chains is calculated for 8‐layer R $\left(1\;1\;0\right)$ and M_1_ $\left(0\;1\;1\right)$ slab models. The results are shown in Figure 6 and compared to d_*V*–*V*_ in 2×2×2 R and M_1_ bulk supercells. The M_1_ $\left(0\;1\;1\right)$ surface shows small alternation of the V−V distances in the top layer, but d_*V*–*V*_ converges to the bulk value in the fourth layer. In the topmost layer of the R $\left(1\;1\;0\right)$ slab d_*V*–*V*_ is larger than for the M_1_ $\left(0\;1\;1\right)$ slab model. The structure of the second and third layer is similar to the R bulk, but the fourth layer shows large d_*V*–*V*_ values, similar to the M_1_ bulk. We therefore conclude that the R $\left(1\;1\;0\right)$ surface is not stable with respect to phase change toward the M_1_ phase. Also the M_1_ $\left(0\;1\;1\right)$ surface reconstructs, but only in the outer layers.

Similar trends were found for the other R and M_1_ surfaces. In Table 1 the optimized V−V bond lengths of the R surfaces and surface supercells with the largest alternation are shown. The V−V distances in the R surfaces change by up to 0.1 Å compared to the bulk and show alternations similar to the M_1_ structure.

**Table 1:** Optimized V−V distances with the largest alternation of low‐index R and M_1_ surfaces in Å; sc‐PBE0 results.

Surface	R	M_1_
(110)/(011)	2.736, 2.942	2.911, 3.056
(010)/(010)	2.812, 2.842	2.819, 2.917
(100)/(001)	2.723, 2.861	2.860, 3.166
(001)/(100)	2.769, 2.901	2.852, 3.126
Exp. (bulk)	2.839, 2.839	2.853, 3.134

The V−V bond length alternation is most pronounced in the R $\left(1\;1\;0\right)$ surface. This effect is present but less pronounced in all other surfaces. The reconstruction of the surfaces is possible due to symmetry lowering compared to the bulk. The transformation is not complete because the surface cell parameters are not optimized in the calculations. Therefore, the surface energies and V−V distances are not the same for the corresponding R and M_1_ surfaces.

### 2.2 Mo‐doped VO_2_

Since it is known that doping stabilizes the rutile bulk phase of VO_2_, we investigated this effect for the most stable $\left(1\;1\;0\right)/\left(0\;1\;1\right)$ surfaces. In preliminary calculations, the difference Δ*E*_M1−R_ was calculated for bulk unit cells MV_3_O_8_ where M=Fe, Co, Ni, Mo and W. These calculations were performed with the SCAN functional^[38]^ and the plane‐wave program VASP,^[39]^ for details see Supporting Material Section (S1,S2). In agreement with the literature it was found that all transition metals stabilize the R phase. The long‐range term of this study is to exploit the M_1_‐R phase transition for catalytic reactions. For this purpose, the two phases should have similar stabilities and the activation barrier should be small. For energetic reasons W doping would therefore be most appropriate. However, W doping leads to pronounced structural changes in the bulk. This can be seen in the V−W distances, which show almost no alternation. We therefore decided to use Mo as dopant element in this study since it showed the second least impact on the relative stability of the phases. This dopant even reverts the sign of Δ*E*_M1−R_, but changes the bulk structure to a lesser extent. The V−Mo distances show similar alteration to the V−V distances in the bulk. Therefore, the M_1_ structure is not significantly changed with Mo as dopant.

**Figure 7:**
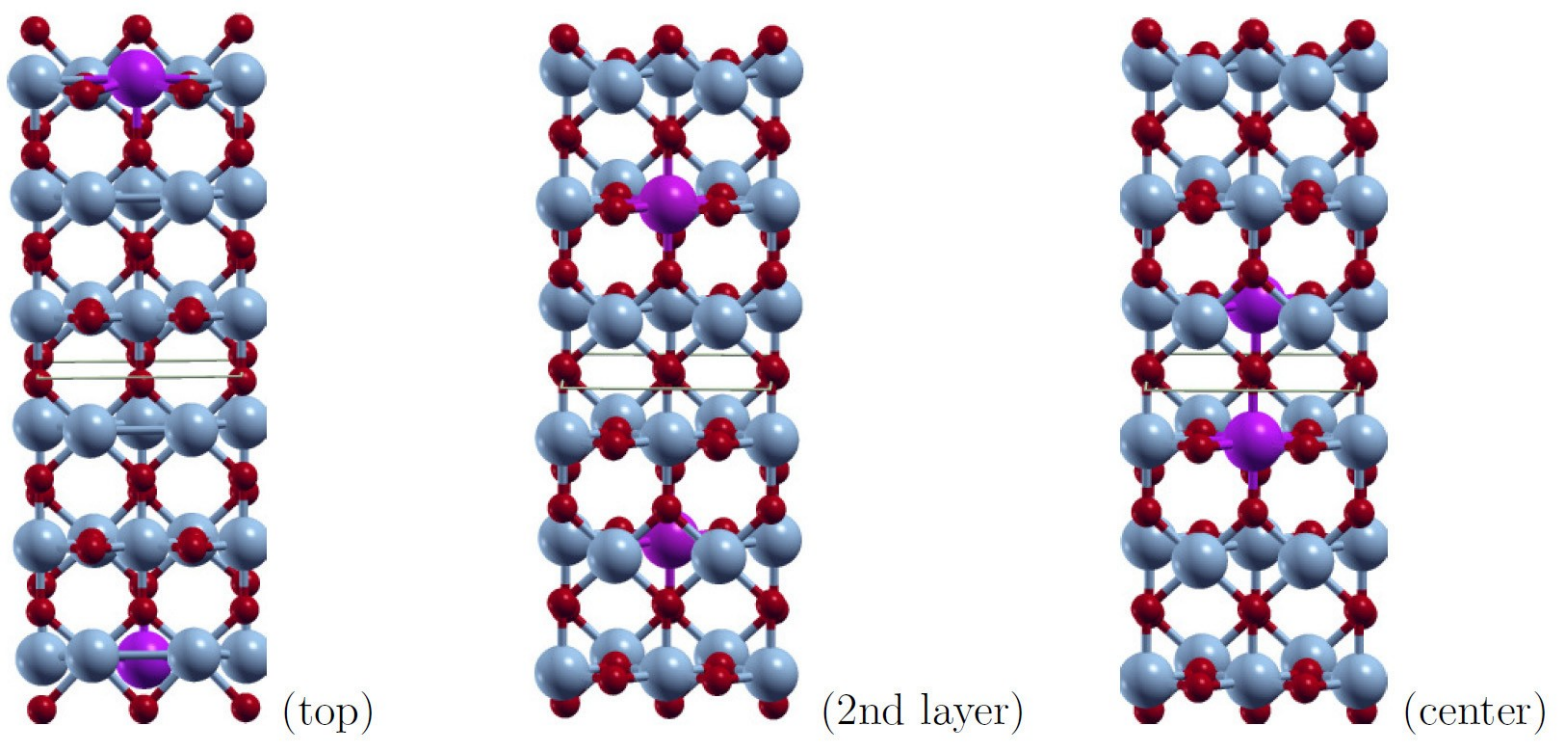
Mo‐dopant (violet) positions shown in the unrelaxed rutile $\left(1\;1\;0\right)$ surface with 6 layers.

We used the same slab models as discussed in the previous section and replaced two symmetry‐equivalent V atoms by Mo. The dopant atoms are placed at three different positions, denoted as *top*, *2nd layer* and *center*, which are shown in Figure 7. The dopant concentration is decreased with increasing number of layers. In this way the effect of dopant concentration of the relative phase stability could be investigated.

**Figure 8:**
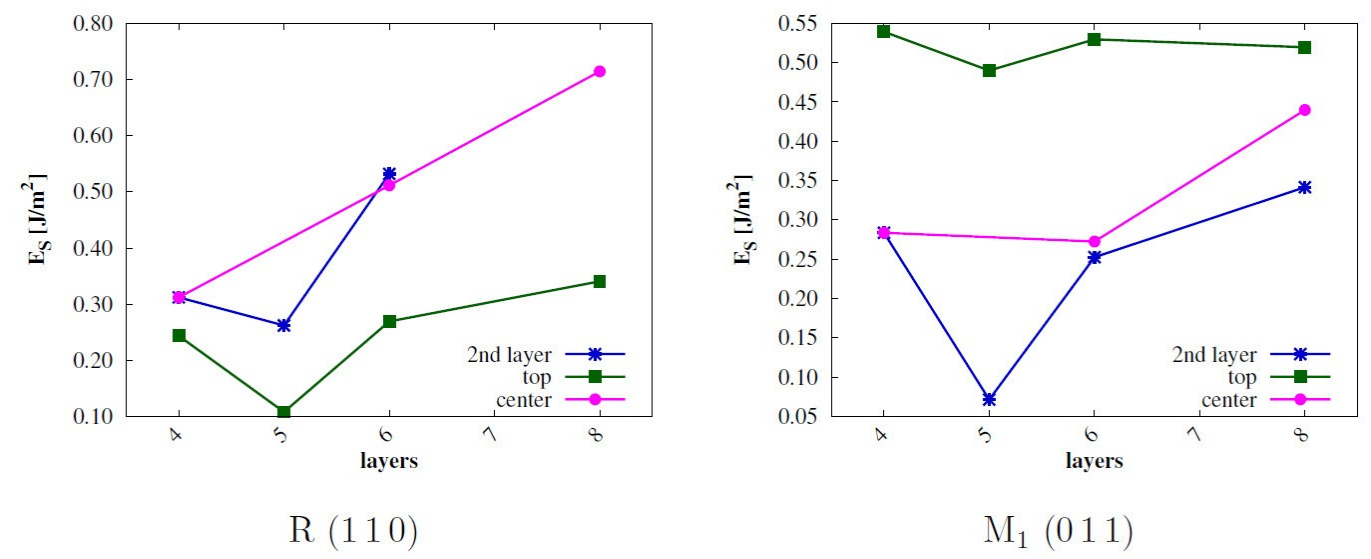
*E_s_* in J/m^2^ of the Mo‐doped R $\left(1\;1\;0\right)$ and M_1_ $\left(0\;1\;1\right)$ surfaces with different V/Mo substitution sites (top, center, 2nd layer) as function of the number of layers *n*; sc‐PBE0 results.

The convergence of the surface energies $E_{s}\left(n\right)$ with the number of layers *n* is shown in Figure 8. The bulk reference in Eqn. 1 is calculated with Mo_2_V_14_O_32_ supercells for both polymorphs. In these supercells the two Mo atoms were placed at maximum distance. In previous theoretical studies it was found that the Mo−Mo distance has only a small effect on the energy^[40]^, therefore we did not vary the dopant configurations.

**Figure 9:**
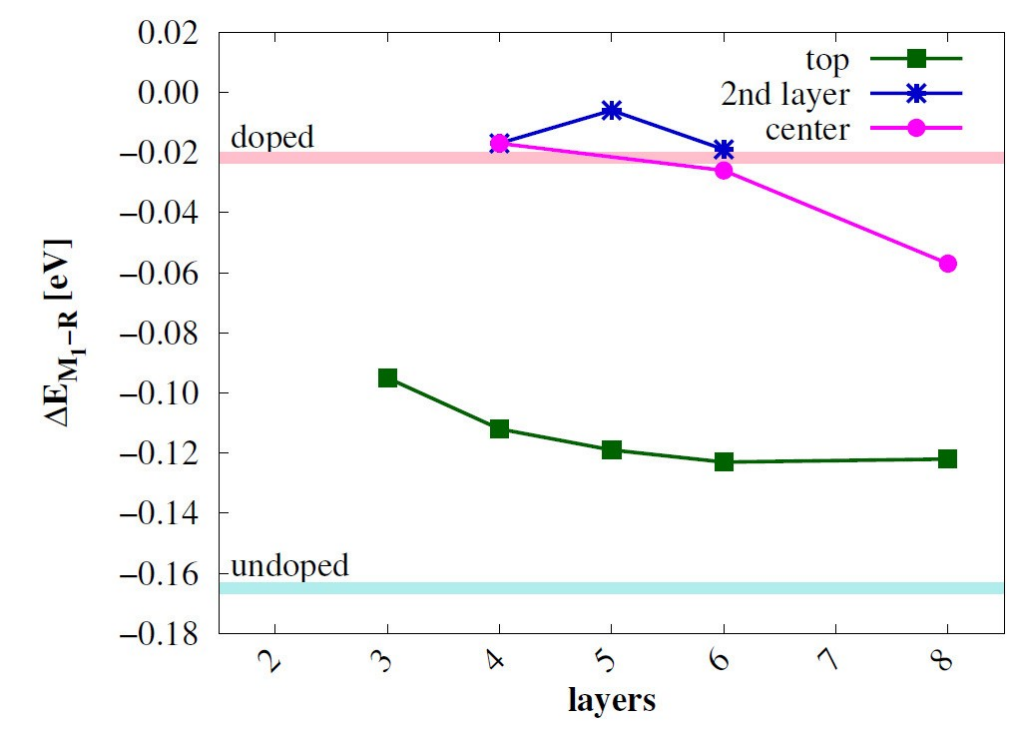
Relative energy of the Mo‐doped R $\left(1\;1\;0\right)$ and M_1_ $\left(0\;1\;1\right)$ surfaces in eV calculated with sc‐PBE0. As reference Δ*E*_M1−R_ of the undoped bulk phases (light blue) and the Mo_2_V_14_O_32_ supercell (pink) are also shown.

In most cases except the *top* doped surfaces *E_s_* does not converge up to n=8 . Larger slab models could not be calculated due to limited computer resources. If Mo is placed at *center* or *2nd layer* positions, *E_s_* increases with the number of layers for both phases. It is expected that only the *top* position is stable for larger models. The relative energy Δ*E*_M1−R_ is calculated for the three Mo positions (Figure 9).

All Mo‐doped surfaces show a stabilization of the R phase in comparison to the undoped bulk. If Mo is placed at the *center* and *2nd layer* position, Δ*E*_M1−R_ is similar to the doped bulk, but convergence is rather slow. For the *top* layer substituted surface Δ*E*_M1−R_=−0.12 eV, in between VO_2_ and Mo_2_V_14_O_32_.

**Figure 10:**
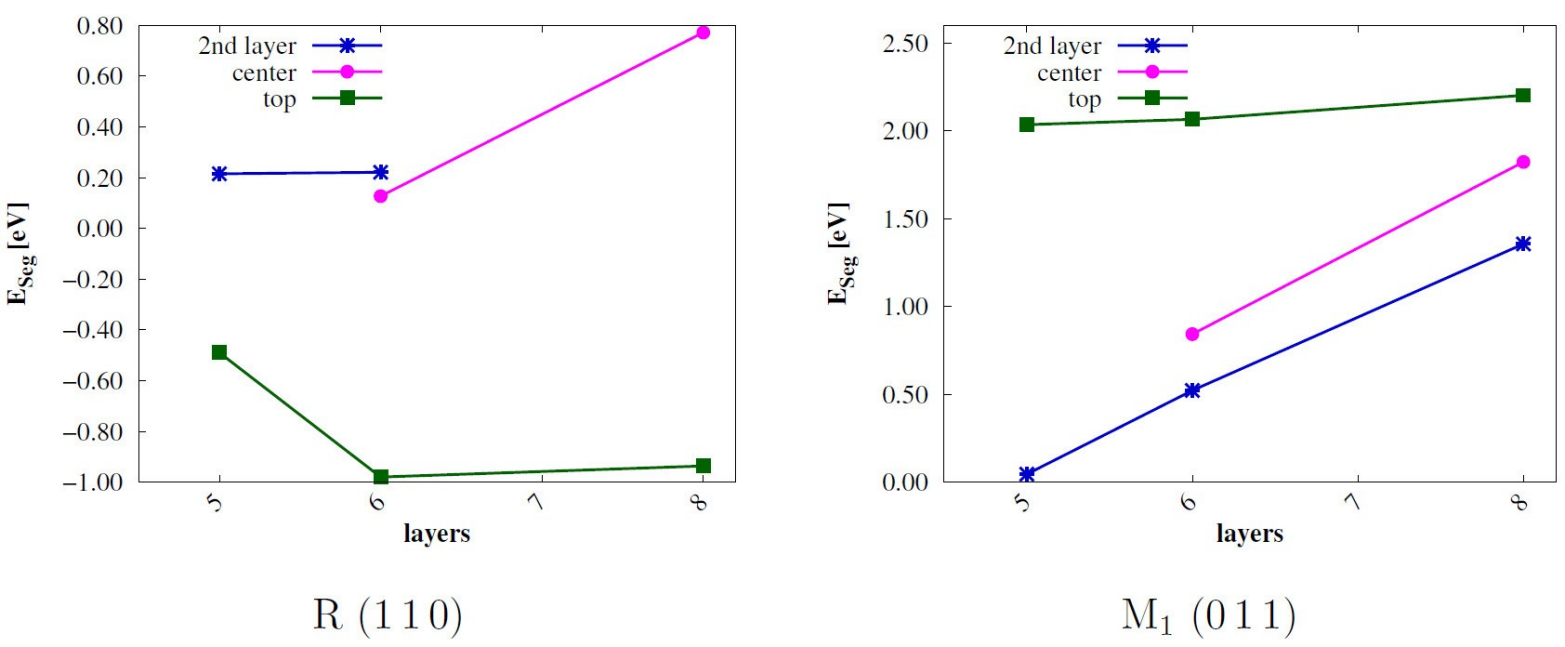
Segregation energy *E*_seg_ in eV of the R $\left(1\;1\;0\right)$ and M_1_ $\left(0\;1\;1\right)$ surfaces with different dopant positions in relation to the number of layers *n*; sc‐PBE0 results.

For further analysis the segregation energies *E*_seg_ are calculated according to Eqn. 2, Figure 10. $E_{\mathrm{bulk},\;\mathrm{doped}}$ and *E*_bulk_ are calculated with MoV_15_O_16_ and V_16_O_32_ supercells of both bulk phases, respectively.

For M_1_ surfaces *E*_seg_ is positive for every Mo position. This means that Mo substitution of the VO_2_ M_1_ $\left(0\;1\;1\right)$ surface is energetically unfavorable. *E*_seg_ increases with the number of layers, only for the *top* position it converges to ≈2.2 eV. At variance, R $\left(1\;1\;0\right)$ surfaces with Mo in the *top* position have a negative E${}_{Seg}\approx =-1$ eV. The other doping positions are energetically unfavorable. For that reason, the top dopant position will exclusively be considered in further research.

**Figure 11:**
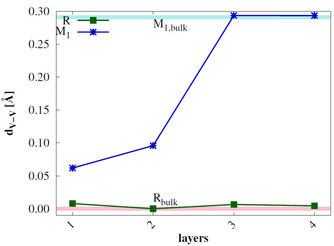
Difference of V−V and V−Mo distances d_*V*–*M*_ of the top doped 8 layer R $\left(1\;1\;0\right)$ and M_1_ $\left(0\;1\;1\right)$ surfaces with the number of layers compared to an undoped 2×2×2 bulk R (pink) and M_1_ (light blue) supercell.

The V−M bond alternation d_*V*–*M*_ is calculated for the 8‐layer top doped R $\left(1\;1\;0\right)$ and M_1_ $\left(0\;1\;1\right)$ slab models similar as for the undoped surfaces. The results are shown in Figure 11 in comparison to the undoped 2×2×2 R and M_1_ bulk supercells. In the top doped R $\left(1\;1\;0\right)$ slab no significant alternation of the V−V and V−Mo distances is observed. The top doped M_1_ $\left(0\;1\;1\right)$ slab shows small bond alternation in the first and second layer. However, the alternation of the V−V distances is larger in the top layer than in the undoped M_1_ surface (Figure 6). The third and fourth layer already show the same V−V bond alternation as the bulk phase. This shows that both the M_1_ and R surface structures are stabilized due to Mo doping.

The V−V and V−Mo distances for all dopant positions in the inner layers of the Mo‐doped surfaces are shown in Table 2 and Table 3.

**Table 2:** V−V and V−Mo distances [Å] in the inner layers of the Mo‐doped *n*‐layer R $\left(1\;1\;0\right)$ slabs for the three Mo configurations.

*n*	top		2^nd^ layer		center	
	V–V	V–Mo	V–V	V–Mo	V–V	V–Mo
3	2.839, 2.839	2.839, 2.839				
4	2.843, 2.843	2.839, 2.839	2.839, 2.839	2.839, 2.839		
5	2.863, 2.863	2.839, 2.839	2.839, 2.839	2.839, 2.839		
6	2.846, 2.842	2.839, 2.839	2.839, 2.839	2.839, 2.839	2.861, 2.839	2.841, 2.841
8					2.839, 2.840	2.839, 2.839
bulk			2.839	2.839

**Table 3:** V−V and V−Mo distances [Å] in the topmost layer of the Mo‐doped n‐layer M_1_ $\left(0\;1\;1\right)$ slabs for the three Mo configurations.

*n*	top		2^nd^ layer		center	
	V–V	V–Mo	V–V	V–Mo	V–V	V–Mo
3	2.939, 3.101	2.804, 3.193				
4	2.965, 3.004	2.963, 3.044	2.994, 2.998	2.988, 2.987		
5	2.981, 2.979	2.825, 3.174	2.907, 3.059	2.984, 2.987		
6	2.944, 3.025	2.961, 3.045	2.934, 3.033	2.951, 3.019	2.903, 3.078	2.986, 2.990
8	2.837, 3.130	2.973, 3.035	2.872, 3.095	2.987, 2.986	2.976, 3.003	2.888, 3.106
bulk			2.853	3.134

In Tables 2 and 3 the V−M bond alternation is analyzed for those layers which showed significant changes in the undoped models. Different from undoped VO_2_, the V−V distances in the inner layers of Mo‐doped R $\left(1\;1\;0\right)$ slabs do not show significant alternation, even with small concentrations of the dopant, 8.3–6.3 % in the 6‐ and 8‐layer models (V−Mo and V−V distances are similar). Therefore, Mo‐doping stabilizes the structure of the rutile surface.

**Figure 12:**
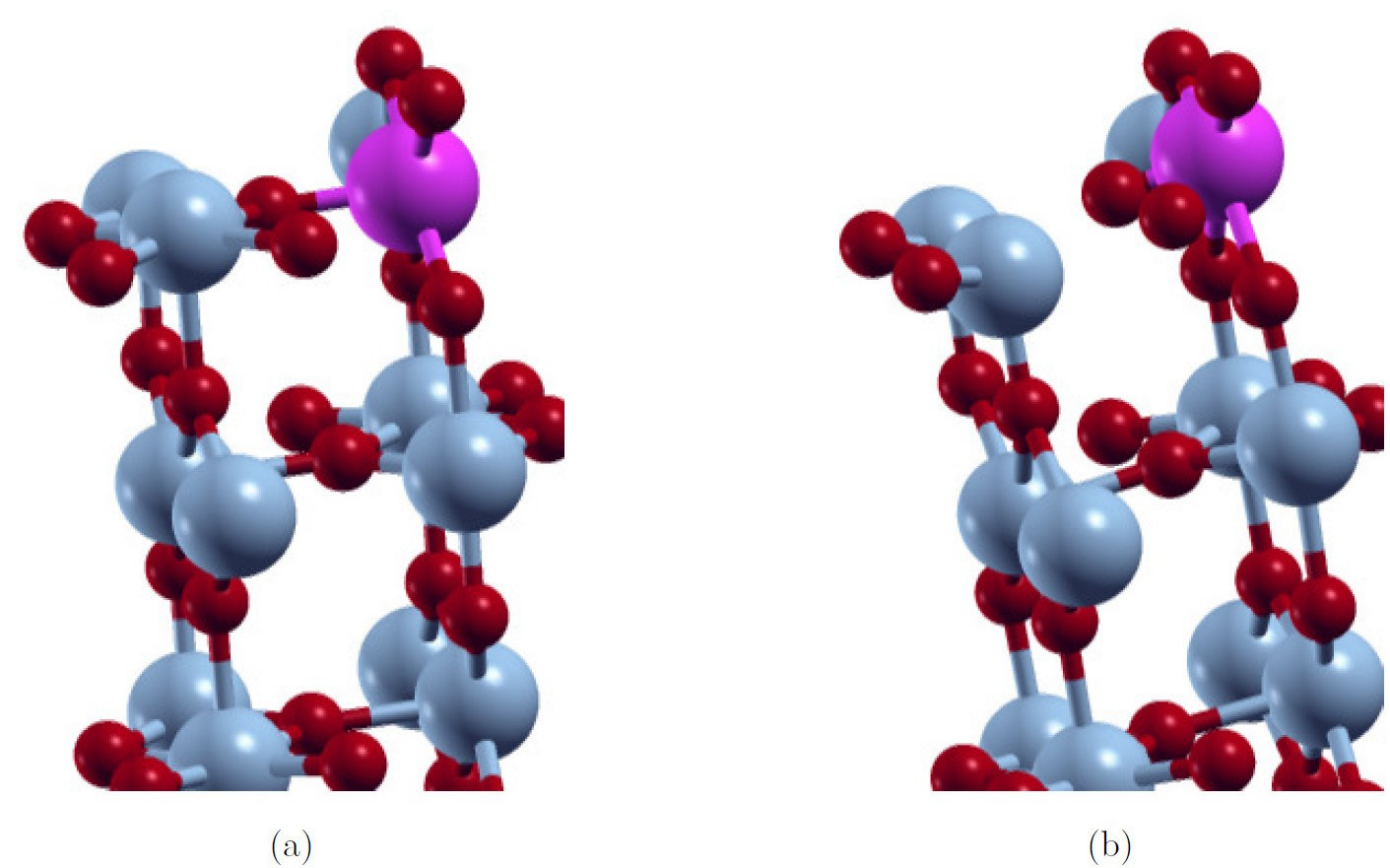
Structure of the relaxed (b) and unrelaxed (a) 8‐layer top doped M_1_ $\left(0\;1\;1\right)$ surfaces; sc‐PBE0 results.

**Figure 13:**
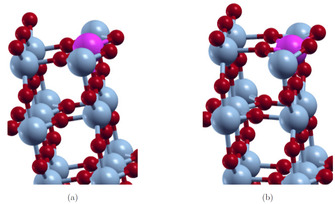
Structure of the relaxed (b) and unrelaxed (a) 8‐layer top doped R $\left(0\;1\;1\right)$ surfaces; sc‐PBE0 results.

Additionally, the effect of the Mo‐dopant on the local atomic structure in the M_1_ $\left(0\;1\;1\right)$ and R $\left(1\;1\;0\right)$ phases is investigated. The unrelaxed and relaxed top doped 8‐layer slab models are shown in Figure 12–13. In both phases, the MO_6_ octahedral structure is distorted due to the Mo‐dopant. The octahedra in the layer with the Mo‐dopant are compressed, while the octahedra in the next layer are expanded. Furthermore, the Mo−O distances are shortened and the octahedra are tilted. These effects are more pronounced in the M_1_ phase.

The topmost layer of the M_1_ $\left(0\;1\;1\right)$ slab shows significant V−V bond alternation, in particular for higher Mo contents (3–6 layers) and top doping. On the other hand, the alternation of the V−Mo distances decreases with the number of layers, except for the *center* Mo position.

In all cases both the Mo‐doped R and M_1_ surfaces are stable and keep the characteristics of the respective bulk structures.

The electronic band gaps and energies of the highest occupied (HOCO) and lowest unoccupied (LUCO) crystalline orbitals, respectively the Fermi energy for metallic cases, of undoped and doped R $\left(1\;1\;0\right)$ /M_1_ $\left(0\;1\;1\right)$ surfaces are shown in Tables S4, S5 and S6 in the Supplementary Material Section. The R $\left(1\;1\;0\right)$ surface is metallic for almost all doped surface models. The structural transition induces a band gap in the undoped R $\left(1\;1\;0\right)$ surface models. The doped M_1_ $\left(0\;1\;1\right)$ surface has a band gap of around 0.4–0.5 eV. For the undoped M_1_ $\left(0\;1\;1\right)$ surface an odd‐even oscillation of the band gap is observed. In average values are ≈0.4 eV larger than for the Mo‐doped surfaces.

The absolute values of the HOCO and LUCO, and Fermi energies should be taken with care since we did not add ghost layers above the surfaces which have been shown to significantly affect the convergence behavior and absolute band positions.^[41]^ As a general trend we observe an upshift of the HOCOs, a downshift of the LUCOs and subsequently a decrease of the band gap due to Mo substitution in M_1_ $\left(0\;1\;1\right)$ surfaces, and an upshift of the Fermi energy for R $\left(1\;1\;0\right)$ (Mo in top position).

Another important property to consider is the effect of doping on the spin population at the V‐atoms and Mo‐atoms as well as their oxidation state. In Table 4 the Mulliken spin populations of the V and Mo atoms of the doped 6‐layer models are shown. In the M_1_ surface with Mo in *top* position and the R surfaces with Mo in *center* and *2nd layer* position, the Mo spin density is close to zero and the V atoms have spin populations larger than 1.0. In the R surface (Mo in *top* position) as well as the M_1_ surface with the dopant in *2nd layer* position the V and Mo spin density is close to 1. The oxidation states of Mo and V are estimated based on the spin population. In the *top* doped R surface as well as the *center* and *2nd layer* doped M_1_ surfaces we conclude that Mo is in the oxidation state +5. In these M_1_ surfaces V atoms close to Mo are partially reduced to V^3+^. The *top* doped R surface and the *center* doped M_1_ surface further show one V‐atom in a 5+ state. The surrounding V‐atoms are partially reduced. In the *center* and *2nd layer* doped R surfaces as well as the *top* doped M_1_ surface Mo is close to 6+. The surrounding V atoms are reduced. Mo^6+^‐atoms were also experimentally found to be present in doped VO_2_ thin films,^[15]^ in particular in the top layers.^[42]^

**Table 4:** Mulliken spin population of the V and Mo atoms of the 6‐layer R $\left(1\;1\;0\right)$ and M_1_ $\left(0\;1\;1\right)$ surfaces; sc‐PBE0 results.

	1^st^ layer				2^nd^ layer				3^rd^ layer			
top	V	Mo	V	V	V	V	V	V	V	V	V	V
R	1.21	1.04	1.20	1.20	1.13	1.18	1.51	1.51	1.20	0.49	1.40	1.40
M_1_	1.24	0.13	1.85	1.18	1.25	1.74	1.19	1.25	1.15	1.13	1.11	1.13

center	V	V	V	V	V	V	V	V	V	Mo	V	V
R	1.13	1.21	0.69	0.69	1.12	1.18	1.50	1.50	1.74	0.51	1.50	1.50
M_1_	1.16	0.23	1.21	1.15	1.17	1.26	1.19	1.72	1.27	1.12	1.28	1.69

2nd layer	V	V	V	V	V	Mo	V	V	V	V	V	V
R	1.26	1.15	1.21	1.21	1.63	0.55	1.48	1.48	1.08	1.11	1.26	1.26
M_1_	1.22	1.09	1.09	1.21	1.20	0.97	1.88	1.20	1.14	1.11	1.10	1.14

**Figure 14:**
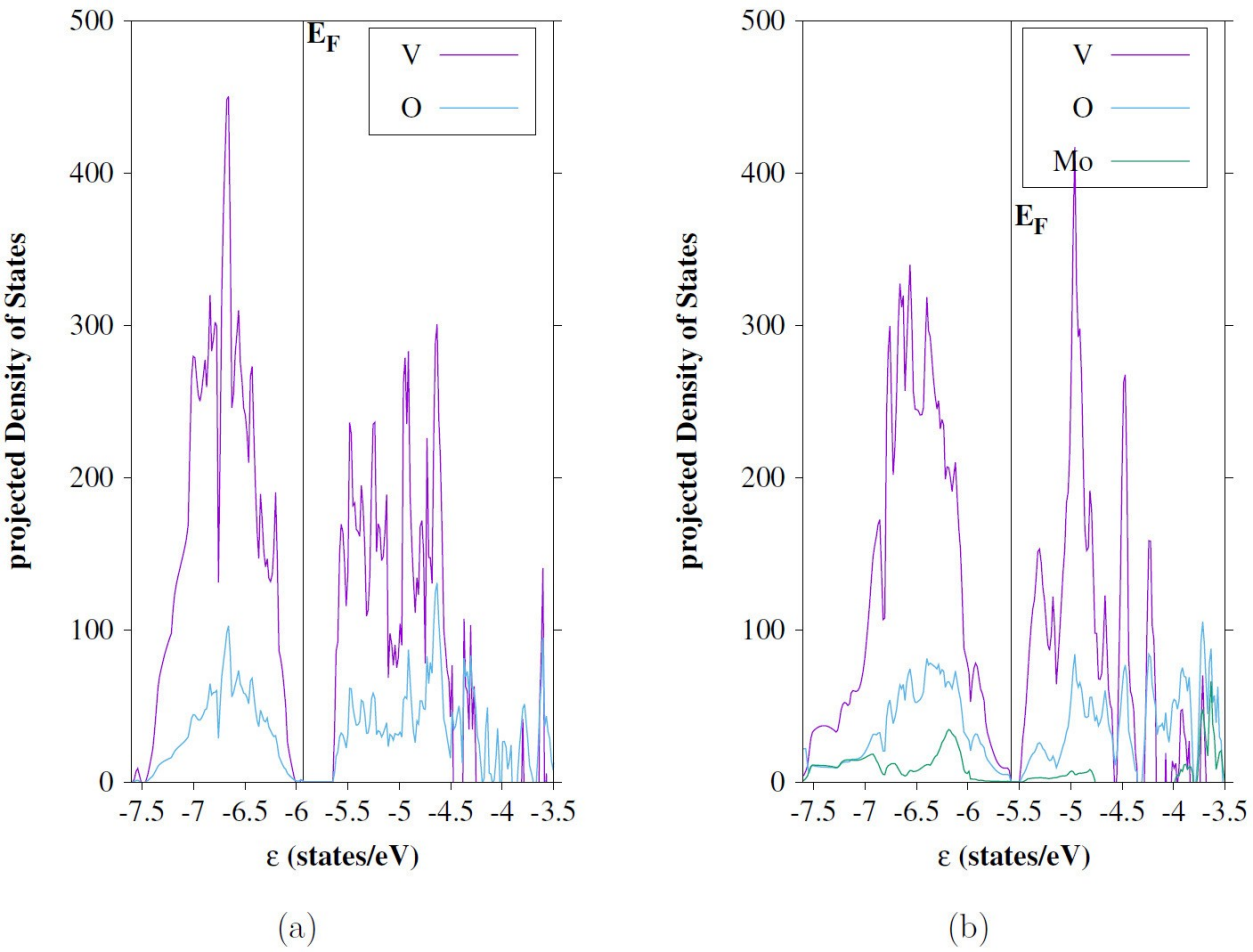
Projected Density of States (pDOS) of the 6‐layer R $\left(1\;1\;0\right)$ surface without (a) and with Mo in top position (b); orbital energies with respect to vacuum level, sc‐PBE0 results

**Figure 15:**
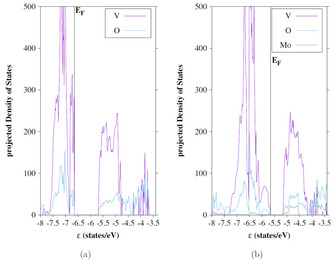
Projected Density of States (pDOS) of the 6‐layer M_1_ (0 1 1) surface without (a) and with doping in top position (b); sc‐PBE0 results.

The projected densities of states (pDOS) have been calculated to further examine the influence of the dopant on the electronic structure. For this purpose, the pDOS of the undoped 6‐layer R $\left(1\;1\;0\right)$ /M_1_ $\left(0\;1\;1\right)$ surfaces are compared to the pDOS of the surfaces doped in top position (Figures 14 and 15). Only the spin‐up electrons are shown in the pDOS. As discussed above an upwards shift of the valence band maximum is observed for the Mo‐substituted surfaces, although the Mo orbitals have only small contributions in the VB. This is consistent with the low spin density at Mo (Table 4). The Mo contributions are slightly larger in the conduction bands. The characteristic localized V d‐states at the Fermi level^[43]^ are diminished in the doped surfaces.

To investigate the influence of the Mo‐dopant atoms on the bonding of the phases the Crystal Orbital Hamilton Population (COHP)^[44]^ is calculated for the 6‐layer *top* doped R $\left(1\;1\;0\right)$ /M_1_ $\left(0\;1\;1\right)$ surfaces.

**Figure 16:**
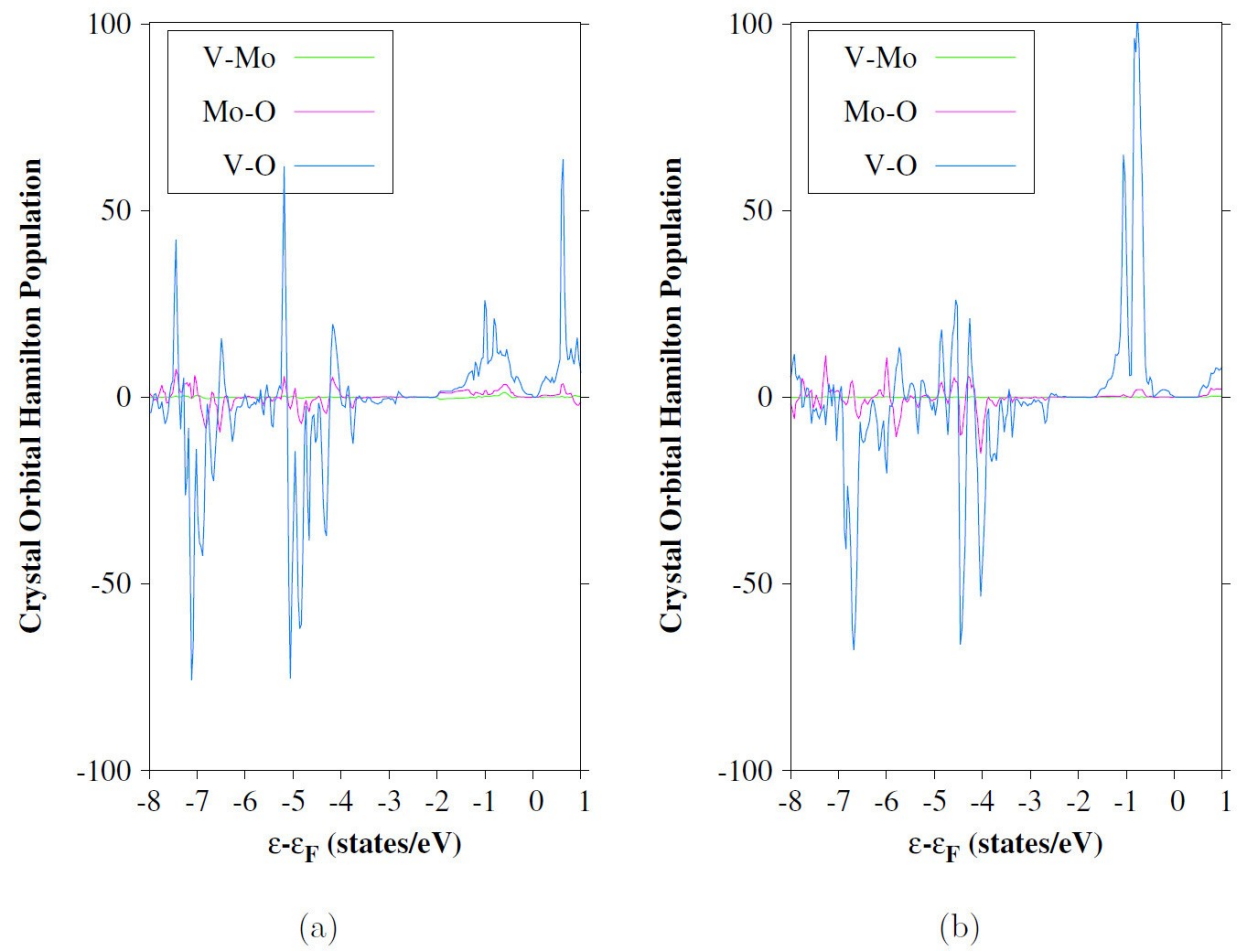
Crystal Orbital Hamilton Population (COHP) of the 6‐layer R (1 1 0) (a) and M_1_ (0 1 1) (b) surfaces with doping in top position; orbital energies are given relative to the Fermi level (eV); sc‐PBE0 results.

Only the V−Mo, Mo−O and V−O interactions are analyzed (Figure 16). Both phases show almost no V−Mo interactions. The COHPs do not show significant differences in the V−Mo or Mo−O bonding. The M_1_ $\left(0\;1\;1\right)$ surface shows more V−O antibonding states compared to the R $\left(1\;1\;0\right)$ surface. Therefore, these states are likely the reason for the destabilization of the M_1_ $\left(1\;1\;0\right)$ surface in comparison to the R $\left(1\;1\;0\right)$ surface.

## 3 Discussion & Outlook

Structural, energetic and electronic properties of the low‐index VO_2_ R and M_1_ phase surfaces are calculated with the self‐consistent hybrid functional sc‐PBE0. In agreement with previous theoretical studies it was found that the R $\left(1\;1\;0\right)$ and M_1_ $\left(0\;1\;1\right)$ surfaces are most stable. However, negative surface energies indicate that the R surfaces reconstruct. In the optimized structures the V−V distances of inner layers alternate similar as in the M_1_ phase. On the other hand, in the topmost layers of M_1_ $\left(0\;1\;1\right)$ surface models the V atoms are equidistant as in the R phase. This would mean that surfaces of R and M_1_ VO_2_ have similar structures, which prevents them from being used as phase‐change catalysts. Upon V/Mo substitution VO_2_ the R $\left(1\;1\;0\right)$ surface is stabilized and no significant V−V and V−Mo bond alternation is observed. Also the V−V distances in the M_1_ phases surfaces are more bulk‐like than in the undoped slabs so that the surface structures of the two phases are clearly distinct. The energy difference between R $\left(1\;1\;0\right)$ and M_1_ $\left(0\;1\;1\right)$ is decreased which should facilitate a temperature‐controlled phase transfer. The segregation energy shows that doping is energetically unfavorable except if Mo is in top layers of the R $\left(1\;1\;0\right)$ surface. V/Mo substitution lowers the band gap and upshifts the valence band maximum of M_1_ $\left(0\;1\;1\right)$ respectively the Fermi level of R $\left(1\;1\;0\right)$ . By an analysis of the spin populations we conclude that the Mo atoms are mostly Mo^V^ and Mo^VI^. Neighboring V atoms are partially reduced to V^III^.

In forthcoming studies the catalytic properties of the R and M_1_ surfaces will be investigated.

## Conflict of interest

The authors declare no conflict of interest.
